# Starch Modification by Organic Acids and Their Derivatives: A Review

**DOI:** 10.3390/molecules201019554

**Published:** 2015-10-27

**Authors:** Đurđica Ačkar, Jurislav Babić, Antun Jozinović, Borislav Miličević, Stela Jokić, Radoslav Miličević, Marija Rajič, Drago Šubarić

**Affiliations:** 1 Department of Food Technology, Faculty of Food Technology, Josip Juraj Strossmayer University of Osijek, Franje Kuhača 20, Osijek 31000, Croatia; E-Mails: dackar@ptfos.hr (Đ.A.); ajozinovic@ptfos.hr (A.J.); bmilicevic@ptfos.hr (B.M.); sjokic@ptfos.hr (S.J.); marija.rajic@ptfos.hr (M.R.); dsubaric@ptfos.hr (D.Š.); 2 Faculty of Technology Tuzla, University of Tuzla, Univerzitetska 8, Tuzla 75000, Bosnia and Herzegovina; E-Mail: radoslavmilicevic@hotmail.com

**Keywords:** starch, acetic anhydride, succinic anhydride, OSA, fatty acids, dicarboxylic acids

## Abstract

Starch has been an inexhaustible subject of research for many decades. It is an inexpensive, readily-available material with extensive application in the food and processing industry. Researchers are continually trying to improve its properties by different modification procedures and expand its application. What is mostly applied in this view are their chemical modifications, among which organic acids have recently drawn the greatest attention, particularly with respect to the application of starch in the food industry. Namely, organic acids naturally occur in many edible plants and many of them are generally recognized as safe (GRAS), which make them ideal modification agents for starch intended for the food industry. The aim of this review is to give a short literature overview of the progress made in the research of starch esterification, etherification, cross-linking, and dual modification with organic acids and their derivatives.

## 1. Introduction

Starch is a naturally occurring, inexpensive polymer with extended application in the food and processing industry due to its safety, biodegradability, inexpensiveness [1], and specific technological properties, such as gelling, thickening, film forming, fat mimicking, *etc.* [2].

Therefore, it is not surprising that starch modifications have attracted thoughtful attention of the scientific community for many years. Numerous original articles regarding a starch modification by chemical, physical and/or enzymatic procedures have been published and starch has been extensively reviewed from different points of views. Novel reviews published within the last five years have mainly dealt with starch digestion and resistant starch [3,4,5,6,7], although physical [8] and chemical modifications have been reviewed as well [9,10,11]. The rheological and pasting properties and biodegradability of starch blended with different components have also been discussed in novel reviews [12].

In 2012 Kaur *et al.* [13] reviewed the progress in starch modifications in the period 2002–2012. They overviewed starch esters and dual modified, physically and enzymatically-modified starches produced in this period and gave insight into their genetic/biotechnology modification. Sweedman *et al.* [10] reviewed octenyl succinic anhydride (OSA)-modified starches in detail from the respective modification methods to their properties.

Although starch has been reviewed in many aspects, and even though many reviews dealing with chemical modifications of starch have been published to date, the application of organic acids and their derivatives in starch modification has not been discussed in detail to the authors’ knowledge. This article will give a short review of the research and advancements regarding the application of organic acids and their derivatives in starch modification. Although the authors have tried to focus on the 2002–2015 period of research, some articles published prior to 2002 are also considered, due to their valuable contribution to the issue.

## 2. Acetylation

Starch acetates are additives approved in the food industry under number E1420. They are commonly produced with acetic acid and acetic anhydride as starch esterification reagents. In addition, vinyl acetate can also be used for esterification.

Concerning the reaction of the above-mentioned reagents with starch, part of hydroxyl groups on anhydro-glucose units is substituted with acetyl groups and, consequently, esters (starch acetates) are formed. The number of acetyl groups incorporated into the starch molecule is dependent on the reactant concentration, pH, reaction time, and presence of catalysts.

### 2.1. Influence of Reactant

Bello-Perez *et al.* [14] have produced highly-substituted barley starch acetate with a reported degree of substitution (DS) of 0.9 by a reaction of 8 mL acetic anhydride per 1 g of starch in aqueous conditions, with NaOH as the catalyst and the reaction time of 30 min.

An increase in the reactant concentration positively influences the reaction efficiency. Namely, the reaction of starch with acetic anhydride in an aqueous medium with NaOH as the catalyst, results in a low degree of substitution, averaging from 0.01 to 0.2 [1], depending on the reactant concentration, starch botanical origin, pH, *etc.* [14]. Babić *et al.* [15] have obtained acetylated corn starch with the DS of 0.05 using 4% acetic anhydride (starch d.w.), with the DS of 0.07 using 6% acetic anhydride and the DS of 0.08 with 8% acetic anhydride. The reaction took place in an aqueous medium with NaOH as the catalyst. In regard to the same reagent concentrations and reaction conditions, these authors have reported the DS of 0.041–0.076 for acetylated tapioca starches [2]. In addition to the reagent concentration, the reagent type also influences the reaction efficiency. Diop *et al.* [16] have acetylated starch with different ratios of acetic acid and acetic anhydride, with iodine as the catalyst and microwave assistance. The DS of the starch modified with acetic acid: acetic anhydride ratios 2:1 and 1:2 were exactly the same (2.656 for 120 mmol acid and 60 mmol anhydride and *vice versa*). However, the highest DS (2.934) was obtained with the 1:1 mixture, although total mmols were kept constant (180 mmol) and the lowest DS (1.837) was obtained when only anhydride was used. These results indicate that the reagent type does indeed influence the reaction efficiency.

The appertaining synthesis routes significantly influence starch acetylation, too, e.g., when producing highly-substituted starch acetate, the addition time of activator potassium carbonate is of great importance—it should be added after acetic anhydride is allowed to penetrate into the starch granules [17].

### 2.2. Influence of Catalysts

The reaction of acetic anhydride with starch favourably yields C-3 and C-6 esters [18]. However, catalysts may influence the type of acetates which are to be generated. The catalysts commonly used in this context include NaOH [15] and H_2_SO_4_ [2]. The base activates starch by forming starch alcoxide (ST-O^−^), which reacts with acetic anhydride to build a starch acetate and NaOAc.

Dicke [18] has reported that the reaction of starch with acetic anhydride with dimethylaminopyridine/pyridine as the catalyst in DMSO results in 50% C-6 esters, 37% of C-2 esters, and 13% of C-3 esters. On the other hand, when disodium hydrogen phosphate was used as the catalyst, more C-2 esters were obtained (69%) at the expense of C-6 and C-3 esters, which emerged in the shares of 20% and 11%, respectively. According to the same author, neutral, weak acid or alkaline salts result in the exclusive formation of C-2 esters, and alkaline catalysts favor formation of C-2 with C-6 and C-3 esters formed in lesser amounts, while acids are not region-selective catalysts.

If acetylation is performed with vinyl acetate as the acetylating agent, C-2 esters are exclusively produced [18].

### 2.3. Starch Granule Properties

Guan *et al.* [19] have reported that the starch granule size influences the degree of substitution as well as the degree of crystallinity. Hence, it is more difficult for acetyl groups to replace OH groups in larger granules and in the crystalline regions where starch chains are highly ordered. Shogren [20] has reported that pre-gelatinized starch reacts with acetic anhydride more rapidly than its native counterpart.

### 2.4. Reaction Time, Temperature, and Medium

Reaction time is an important factor for the acetylation efficiency. Han *et al.* [21] have revealed that the DS increases with the reaction time which rose from 60 to 150 min, after which the DS decreases with reaction extension to 240 min due to hydrolysis of starch acetate.

A temperature increase from 25 to 30 °C facilitates diffusion of acetylating agents and starch swelling, which results in higher yields of a substituted product. However, acetylation is an exothermic reaction and a further increase of the temperature would negatively impact the reaction [21].

However, Chi *et al.* [1] have documented a DS increase from 0.81 at 50 °C to 2.83 at 75 °C with acetylated corn starch produced by a reaction with glacial acetic acid and acetic anhydride in the presence of methane sulphonic acid.

Water is a commonly used reaction medium for acetylation. A higher water content aids dissociation, diffusion, and adsorption of the esterifying agent, which is favorable to the reaction. However, if the water to starch ratio exceeds 1.06:1, the reaction efficiency is reduced due to side reactions [21].

Since a high water concentration is required to avoid mixing problems in industrial conditions, acetic anhydride hydrolyzes to acetic acid, which reduces the reaction efficiency. To overcome this problem, pyridine and DMSO can be used as solvents [22]. In addition to the high environmental impact of these solvents, the drawback of DMSO refers to a difficult separation of the solvent from starch [23].

A possible solution for these problems can be found in the research of Muljana *et al.* [22] who have proposed densified CO_2_ as a “green” solvent for low substituted starch acetate production. They have obtained potato starches substituted with acetic anhydride using NaOH as the catalyst with a range of the DS between 0.01 and 0.46, showing a high potential of the densified CO_2_ usage as a solvent medium. In addition, Shogren [20] has reported that surface acetylation of starch with acetic acid could be performed even in anhydrous conditions at 80 °C, using scandium triflate as the catalyst.

### 2.5. Starch Acetate Properties and Applications

Since an acetyl group is much bulkier than a hydroxyl group, it sterically hinders the structural organization of starch chains. Due to the repulsion between starch molecules, water percolation between chains is facilitated [24]. Thus, the swelling power and solubility of starch are increased and less energy is required for gelatinization, which results in a lower gelatinization temperature and enthalpy. A starch retrogradation is also influenced by this phenomenon—due to the steric hindrance, starch chains are less prone to form hydrogen bonds and re-associate and acetylated starches are, therefore, less prone to retrogradation [15,25].

Sodhi and Singh [26] have registered a decrease of the pasting temperature and an increase of the peak and cold viscosities after acetylation of rice starches. Similar results have been obtained by Liu *et al.* [27] after acetylation of maize starches. The introduction of an acetyl group causes weakening of the molecular structure by interrupting the hydrogen bonds and the helical structure in the starch granule, resulting in a decreased pasting temperature and an increased breakdown [28]. However, Muhamedbegović *et al.* [25] have observed a decrease of the viscosities after acetylation of potato starch and similar observation has been reported by Berski *et al.* [29] for acetylated oat starch and Saartrat *et al.* [30] for acetylated canna starch. Huang *et al.* [31] have postulated that the viscosity of starch pastes could be influenced by the homogeneity of acetylation, e.g., whether it is limited to the outer lamellae of granules or located in their inner part. Saartrat *et al.* [30] have demonstrated that the viscosity of acetylated starches is influenced by two factors: a weakening starch granule due to disruption of the inter- and intra-molecular bonds, and reduced bonding with water molecules due to the hydrophobicity of acetyl groups. Depending on the interplay between these two factors, the viscosity could be decreased or increased by acetylation.

Starch paste clarity and freeze-thaw stability are increased by starch acetylation [2,26]. Due to the high content of hydrophobic acetyl groups, high DS acetylated starches develop hydrophobicity [32] and thermoplasticity and are soluble in acetone and chloroform [16]. Starch acetates with a low DS are commonly used in the food industry as consistency, texture, and stability improvers [14] and, according to the FDA, the maximum DS of acetylated starches for food application is 0.2 [21].

Starch acetates with a higher DS appear in extensive non-food applications, such as tablet binders, cigarette filters, biodegradable packaging materials, coatings, adhesives, *etc.* (Table 1) [1].

**Table 1:** Summary of starch modifications with organic acids and their derivatives.

Modification Type	Advantages	Disadvantages	Application	Source
Acetylation	Improved paste clarity	Instability during shearing at high temperatures	*Food industry:*	[14,15,17,21,26,30]
	Retarded retrogradation		Consistency, texture and stability improvers	
	Decreased gelatinization temperature		*Non-food application:*	
	Freeze-thaw stability		Tablet binders, cigarette filters, biodegradable packaging materials, coatings, adhesives *etc.*	
Succinylation	Starch solubility in cold water	Instability during shearing at high temperatures	Thickening or stabilizing agents in soups, snacks and frozen food products	[33,34,35,36,37,38]
	High viscosity			
	Better thickening power			
	Improved paste clarity			
	Retarded retrogradation			
	Freeze-thaw stability			
OSA * modification	Decreased gelatinization temperature		Beverage emulsion stabilizers	[10,32,39,40,41]
	Increased paste viscosity Ability to stabilize water/oil emulsions		Encapsulation of flavor compounds	
	Reduces glycemic response after consumption of beverages			
SCFA * modification	Nutritional role—prebiotic action	Decreased biodegradability	Potential prebiotic	[42,43,44]
	Increased water resistance			
MCFA * modification	Increased water resistance		Starch/LDPE blends	[44,45]
	Higher stability at high temperatures		Edible films	
LCMA* modification	Increased hydrophobicity	Decreased glass transition temperature	Potential resistant starch Starch films	[46,47]
	Higher resistance to α-amylolysis			
Adipic acid/acetanhydride modification	Higher paste clarity Improved paste stability Higher viscosity values	Lower solubility	Thickening agent	[48,49,50,51,52]
Esterification with ferulic acid chloride	Enhanced hydrophilic properties Improved absorption of ferulic acid in intestine	Poor mechanical properties	Functional food ingredient	[53]
Succynilation	Resistance to enzyme digestibility	Decreased freeze-thaw stability	Resistant starch	[7,54]
Maleate esters	Non-toxicity Biocompatibility Low cost	Promoted hydrolysis and glucosidation reactions	Drug delivery carrier	[55,56,57]

* OSA, octenylsuccinic anhydride; SCFA, short-chain fatty acids; MCFA, medium-chain fatty acids; LCFA, long-chain fatty acids.

## 3. Succinylation

### 3.1. Reaction Mechanisms

**Figure 1:**
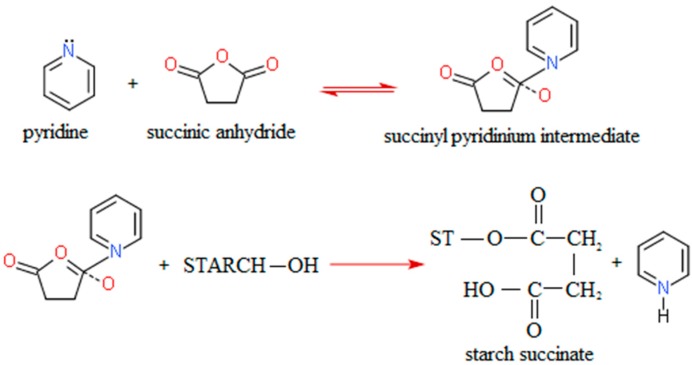
Modification of starch by succinylation [33].

To react with succinic anhydride, starch granules have to be disrupted. Therefore, succinylation of starch with succinic anhydride is performed by refluxing with pyridine at 115 °C or by gelatinization of the starch in aqueous pyridine followed by a reaction with succinic anhydride in 100% pyridine at 115 °C. Pyridine has a dual function in the reaction: it activates starch, making it nucleophillic, and reacts with succinic anhydride to form succinyl pyridinium intermediate. This intermediate reacts with starch generating starch succinate and pyridine (Figure 1) [33].

To achieve a complete reaction with pyridine as the catalyst, a sufficient amount of pyridine has to be available—a starch/pyridine ratio of 1:2 is necessary to assure optimal reaction conditions. This is a limiting factor for industrial application of this modification. Namely, pyridine is a toxic and expensive chemical. It can be recovered from the reaction by distillation after washing starch succinate with water, but this procedure is uneconomic [33].

Another proposed mechanism involves 4-dimethylaminopyridine as the catalyst and DMSO as the solvent at room temperature for 24 h [34]. Chang *et al.* [34] have prepared a succinic ester from porous potato starch by mixing the porous starch with a succinic anhydride solution in acetone and the subsequent reaction at 110 °C for 4 h, whereas Sui *et al.* [58] have induced a reaction by drop-wise adding succinic anhydride to a water suspension of starch with the maintaining pH close to 8.5 by drop-wise adding NaOH.

### 3.2. Reaction Efficiency

The efficiency of a succinylation reaction is influenced by the starch granule size, reagents used, and appertaining reaction time [33]. Chang *et al.* [34] have shown a linear increase in the DS coinciding with an increase of the succinic anhydride/starch ratio due to the greater availability of anhydride molecules for starch granules. Solvent is also an important factor in the reaction efficiency—a significantly higher DS is achieved when DMSO is used as a solvent instead of water [59]. Reaction time is a limiting factor, but only to a certain extent. Namely, Bhandari and Singhal [33] observed a significant DS increase with the prolongation of the reaction time from 1 h to 5 h. Further time increase from 5 h to 10 h did not significantly influence the reaction efficiency.

### 3.3. Starch Succinate Properties and Application

Succinylation of starch leads to addition of hydrophilic negatively charged succinyl groups which in turn impart a hydrophilic character to starch [60]. Succinic groups weaken internal bonding in starch granules and facilitate starch solubilization even in cold water [58]. However, the research of Olayinka *et al.* [35] has revealed an increase of the swelling power after succinylation of sorghum starches, but no significant effect has been determined in the solubility.

Gelatinization temperature and enthalpy are decreased by succinylation of maize starch [58]. Due to the inhibition of the ordered starch structure, the paste clarity is improved and the retrogradation is retarded [36,58]. The latter, however, could be dependent on the starch type according to Olayinka *et al.* [35]. These authors have identified no significant effect of succinylation on the retrogradation of red sorghum starch, while succinylated white sorghum starch had a significantly lower retrogradation enthalpy compared to its native counterpart. Hydrophilic succinic groups prevent syneresis during freeze-thaw cycles [36]. Mehboob *et al.* [60] have shown that the succinylation of both native sorghum and acid-thinned starches reduces retrogradation in starches while the peak, cold paste, and water binding capacity, and setback viscosities are characterized by a sharp rise.

The peak viscosity, final viscosity, breakdown, and setback values go up after succinylation of normal maize starch [58]. The increased setback and breakdown values indicate reduced stability during shearing at high temperatures and cooling. Olayinka *et al.* [35] have demonstrated that these parameters are increased by succinlyation of red sorghum starch and decreased by modification of a white sorghum starch. Rudnik *et al.* [61] have reported thermally-stable potato, corn, and wheat starch succinates, proportional to their DS, up to 200 °C.

Olayinka *et al.* [35] have stated that starch succinates have advantages, such as high viscosity, greater thickening power, low gelatinization, and retrogradation.

Succinylated starches could be useful in a preparation of non-gelling custard creams as they increase the viscosity of the latter. In addition, due to their higher hydrophilicity, they could give more succulent and juicy meat and fried products [60]. Starch succinates are also used as thickening or stabilizing agents in soups, snacks, and frozen/refrigerated food products [37].

## 4. Octenylsuccinic Anhydride (OSA) Modification

### 4.1. Proposed Mechanisms

**Figure 2:**
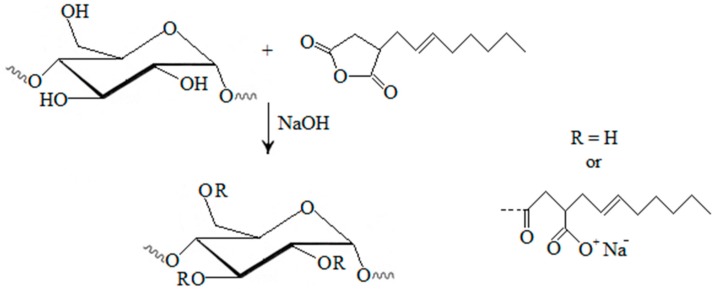
Starches modified with octenyl succinic anhydride [10].

Octenylsuccinic starches are commonly produced by esterification of starch with octenylsuccinic anhydride in aqueous media under alkaline conditions (Figure 2) [10,39]. The reaction of OSA and starch carried out in the aqueous phase results in poor reaction efficiency and uneven distribution of OSA groups [62]. Alternative procedures were conducted with pyridine and acid chlorides, resulting in significant production of byproducts [63]. Xu *et al.* [63] have proposed lipase-coupling esterification as a “green” process which yields a high-quality product. The process includes pre-treatment of a starch suspension at a high temperature before enzyme coupled esterification, which is performed at a constant temperature. Some studies show that OSA groups could go deep into the interior of starch granules and be distributed throughout the starch granules by an ultrasound or hydrothermal treatment [64,65]. Wang *et al.* [62] have proved that high-speed shear conditions can be used to improve the reaction efficiency of starch modification by OSA, and improve the clarity and freeze-thaw stability of modified starches.

The starch-OSA reaction can be improved with pre-treatment of starch with α-amylase, which increases the starch surface and enables the reaction to occur in the inner part of granules [10].

### 4.2. Influence of Temperature

The optimal temperature for a reaction of starch with octenylsuccinic anhydride ranges from 30 to 40 °C, with the peak at 35 °C for amaranth, *Indica* rice, maize, waxy maize, and potato [10]. In terms of the lipase-catalyzed esterification of waxy corn starch, the optimum conditions require starch pre-treatment at 65 °C. Xu *et al.* [63] have postulated that at lower temperatures starch granules are not loosened enough, while at higher temperatures starch completely gelatinizes and creates a viscous medium that is hard to agitate. The maximum lipase concentration up to which the reaction efficiency increases is 0.6%. Above this content, the reaction efficiency is kept constant, due to the catalysis of octenylsuccinic starch hydrolysis [63].

The optimum pH for octenylsuccinylation is 7.0–8.0. At this pH range, starch molecules are activated for a nucleophylic attack. In addition, if the reaction is catalyzed by an enzyme, the pH value optimal for its activity is 8.0 [63]. If the pH exceeds 9.5, undesirable secondary reactions occur and a large quantity of byproducts are produced [66].

Segura-Campos *et al.* [66] have reported that larger amounts of anhydride should be used when preparing the OSA starch to facilitate a synthesis reaction by a larger number of collisions.

### 4.3. OSA Starch Properties and Application

Starch modification by OSA gives a specific surface activity to starch by incorporating hydrophobic alkenyl groups from OSA into the hydrophilic starch molecule which interrupts the linearity of amylose and the ramified portion of amylopectin [67]. Therefore, OSA starches are characterized by a decreased gelatinization temperature, increased paste viscosity and the ability to stabilize water/oil emulsions [66,68].

Octenylsuccinic starches are used as emulsion stabilizers in the molecular form, surface active hydrocolloids, texturizing agents in many food-systems, for flavor compound encapsulation, as well as in pharmaceutical and biodegradable plastic industries [40,67] (Table 1).

According to the research of Heacock *et al.* [69], octenylsuccinic starch reduces the glycemic response after consumption of beverages, which is indicative of potential application of this modified starch in functional foods. Li *et al.* [41] have reported a resistance of OSA-starch to digestion and recommended it as a drug delivery system. However, to assure these, additional extensive research should be conducted.

## 5. Modification with Fatty Acid Derivatives

According to the IUPAC Golden book [70], fatty acids are “aliphatic monocarboxylic acids derived from or contained in an esterified form in animal or vegetable fat, oil or wax. Natural fatty acids commonly have a chain of 4 to 28 carbons”. Fatty acids can be categorized based on a number of carbon atoms into: short-chain fatty acids (SCFA; less than eight carbon atoms), medium-chain fatty acids (MCFA; 8–14 carbon atoms) and long-chain fatty acids (16 carbon atoms or more). All three types have been used in starch modification.

### 5.1. Short-Chain Fatty Acids

Main short-chain fatty acids are acetic, propionic, and butyric acid. Since starch acetylation is a widely applied modification method, it is reviewed as a separate method at the beginning of this article.

Similar to acetylation, starch can be propionated or butyrilated. Santayanon and Wootthikanokkhan [42] have activated cassava starch with pyridine and have allowed the reaction with propionic anhydride to proceed for 22 h. The propionyl content in modified starch varied from 6.8% to 51.1%, depending on the temperature and anhydride quantity used for the modification (8.2–18.2 mL). An interesting observation is that the maximum propionyl content was not achieved with the maximum reagent content, but with 10.2 mL of anhydride. The authors have stated that this could be due to the ester hydrolysis of modified starch. In addition, the biodegradability decreased and moisture resistance increased after the modification.

Lopez-Rubio *et al.* [43] have modified a maize starch with acetic, propionic and butyric anhydride with NaOH as the catalyst. The modification with the DS above 0.2 induces changes both in the crystallinity and at the nanoscale level. More significant structural changes have been observed for the reagents of a smaller molecular weight.

Starch modified with a short-chain fatty acid could have an important role in nutrition. Namely, some studies have shown that these starches deliver SCFAs to the large bowel, thus stimulating the growth of beneficial intestinal microflora. In this way, the intestinal health is improved.

### 5.2. Medium-Chain Fatty Acids

A common esterification reaction is starch modification with octanoyl chloride, with pyridine as a solvent and a catalyst [44,71]. Modified starch has been with low density polyethylene (LDPE). The obtained mixture has higher thermal stability and lower water absorption compared to the plasticised starch/LDPE blends [71]. Another positive effect of starch modification with octanoyl chloride is a decrease of water penetration through the starch film, proportionally to substitution degree [44]. The water resistance of starch has been also increased by transesterification with methyl laurate under the solvent-free conditions [72].

The esterification of starch nanoparticles with octanoyl, nonanoyl, and decanoyl chloride, using water as an environmentally-friendly solvent results in a decreased starch polarity and an increased starch miscibility with chloroform, and a higher stability at high temperatures [45]. Complexation of starch with lauric acid resulted in an increase of pasting and gelatinization temperatures and retarded retrogradation compared to the native starches.

### 5.3. Long-Chain Fatty Acids

Gao *et al.* [46] have esterified starch with palmitic and stearic acid in ionic liquids which disrupted semicrystalline structures of starch granules and have, thus, facilitated esterification reactions. Higher DS values have been obtained for palmitic acid, due to its lower molar mass.

Jimenez *et al.* [47] have prepared starch based films with the addition of palmitic, stearic, and oleic acid. Fatty acids do not influence the sorption behavior of starch films. However, the glass transition temperature decreases with the most prominent effect of palmitic acid derivatization, followed by stearic acid. Oleic acid does not significantly influence the glass transition temperature. Saturated fatty acids induce a slight loss of the matrix strength, while oleic acid incorporation provoked a much greater reduction of rigidity.

Kapusniak and Siemion [73] have investigated the reaction of potato starch with long-chain fatty acids. The reaction of starch with oleic acid on conventional heating results in a formation of ester, while microwave heating induces a microencapsulation of oleic acid in starch granules. The reaction of starch with linoleic acid on conventional heating results in an esterification, too. Obtained starch esters were more hydrophobic and more resistant to α-amylolysis.

## 6. Modification with Dicarboxylic Acids

### 6.1. Adipic Acid

**Figure 3:**
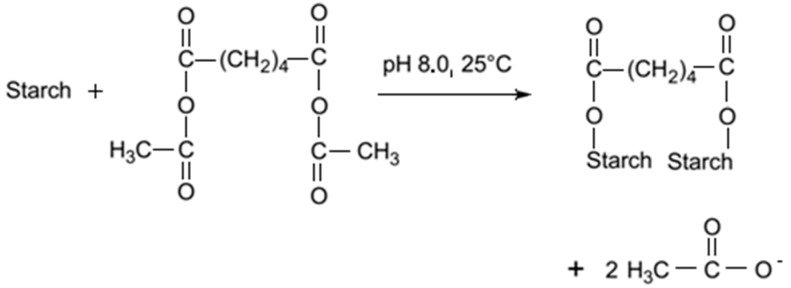
Cross*-*linking of starch with adipate [74].

Adipic acid is a commonly used reagent for starch modification. Since it has two carboxylic groups, it can produce cross-linked starch, as well as monosubstituted derivatives. The reaction is conducted in a water suspension, in alkali conditions, by the drop-wise addition of acetic anhydride/adipic acid mixture. By mixing acetic anhydride and adipic acid, mixed acetic adipic anhydride is formed. This anhydride reacts with starch producing a distarch adipate and acetic acid (Figure 3). At pH 8.0 the reaction is fast, but the reagent has to be slowly added with the pH maintenance close to 8.0 [74].

Distarch adipates are more stable during shearing at high temperatures, at low pH, and in freeze-thaw cycles [48].

#### Modified Starch Properties

Mali and Grossmann [48] have reported that an acetylated distarch adipate produced by an extrusion has higher paste clarity, and during 15 days of storage at 10 °C no syneresis has been recorded. Starch paste stability has also been improved by the reaction of waxy potato starch with the mixture of adipic acid and acetic anhydride [49]. Tapioca, barley, and wheat starches modified with a mixture of adipic acid and acetic anhydride have lower gelatinization temperatures, higher viscosity values, and lower solubility (determined at 95 °C) than their native counterparts [50,51,52].

Kapelko-Zeberska *et al.* [75] have conducted esterification of retrograded potato starch with acetic and adipic acid. The resistance of modified starches to the action of amyloglucosidases has increased along with the increase of the total degree of esterification, as well as the viscosity of the starch pastes. The study demonstrated that acetylated-adipate starches may be classified as a preparation of RS 3/4 type resistant starch with good texture-forming properties (Table 1).

### 6.2. Other Dicarboxylic Acids

Mathew and Abraham [53] have prepared a starch ferulate by esterification with ferulic acid chloride, using DMSO as the solvent and pyridine as the catalyst. They have achieved DS 0.15–0.77, depending on the quantity of ferulic chloride that was used. Modified starch has enhanced hydrophilic and poor mechanical properties. However, the absorption of ferulic acid, which has a high antioxidant activity, in intestine was improved, which implies that a starch ferulate could be used as a functional food.

Kim *et al.* [76] have prepared adley starch glutarate by reacting the starch with glutaric acid in acidic conditions for 16 h at a room temperature. They have reported a decrease of starch digestibility, solubility and gelatinization enthalpy. Heat treatment does not influence the digestibility due to the limited swelling.

Ačkar *et al.* [77] have modified barley starch with a mixture of glutaric acid and acetic anhydride in a water suspension, maintaining pH close to 9.0 with NaOH. They have reported an increase of digestibility, peak viscosity, solubility and swelling power, with a decrease of gelatinization temperature.

Modification of wheat starch with mixtures of acetic anhydride with succinic and azelaic acid, respectively, with NaOH as the catalyst decreases gelatinization and pasting temperatures, as well as a retrogradation after seven and 14 day-storage at 4 °C. The viscosity increases, while the stability during shearing at high temperatures decreases. However, the freeze-thaw stability also decreases in both investigated modifications. Furthermore, the swelling power, solubility, and resistant starch content increase in both investigated modifications. FT-IR analysis of modified starches showed a peak around 1.740 cm^−1^, which is a characteristic of a carbonyl group of ester. The total color difference caused by modifications was detectable only by trained people [7,54].

John and Raja [78] have modified cassava starch with oxalic, malonic, and succinic acid. Compared to the native starch, oxalic, and malonic starch complexes have higher solubility and lower pH due to the larger number of carboxylic groups. The amylose content has been decreased by these modifications. Starch-succinic acid complexes are the most resistant to enzyme digestibility.

Corn and potato starch have been cross-linked with malonic acid (15%–50% starch d.w.) after gelatinization at 90 °C for 45 min [79]. The reaction has been catalyzed with sodium hypophosphite monohydrate. DS values are higher when potato starch is modified—for 25% malonic acid, the DS for potato starch was 0.16, and for corn starch, 0.08. The swelling power decreases with an increase of malonic acid content used for modification due to the higher extent of cross-linking. TGA analysis has shown no changes in thermal properties after the modification.

Biswas *et al.* [55] have prepared starch maleate half-esters using microwave and conventional esterification with maleic anhydride in DMSO, with pyridine as the catalyst. The microwave treatment reduces reaction time and increases reaction efficiency. However, viscosity measurements reveal a partial hydrolysis of glycosidic bonds.

Thermoplastic starch-maleate esters have been prepared by an extrusion, with glycerol as plasticizer, as reported by Raquez *et al.* [56]. FTIR and NMR analyses have shown that maleate groups grafted on the starch backbone promoted hydrolysis and glucosidation reactions.

Tay *et al.* [57] have prepared starch-maleate monoester and cross-linked starch in aqueous conditions, reacting starch with maleic anhydride for 4 h at 100 °C, with NaOH as the catalyst. DS values of obtained modificates ranged from 0.03 to 0.21. The water absorbency and hydrophilicity of monoesters are lower for samples with the lower DS values. Authors have suggested a possible application of the cross-linked starch as a drug delivery carrier, due to their non-toxicity, biocompatibility, and low cost.

## 7. Conclusions

Organic acids are extensively researched in terms of starch modification. Most studies are dedicated to commonly used acids and their derivatives, such as acetic anhydride, succinic anhydride, and OSA (Table 1). However, this literature review has shown that other organic acids and their derivatives have a high potential in the production of modified starches for the food and processing industry.

The variety of modified starches can be obtained not only by the selection of starting starch type, but also by the careful selection of modifying agents, catalysts, reaction temperature, and time. Reactions can be facilitated by the combined application of physical modifications, such as extrusion or pre-gelatinization. The complexity of modification is, therefore, high and by changing only one reaction parameter, it is possible to obtain a new product with significantly different properties. This allows much space for further research on reactions of starch with organic acids.

In addition, a very important aspect is that some of these modified starches should be additionally researched in order to determine their safety for the consumption. On the other hand, a lot of them can already be characterized as good functional food ingredients or good bio-based packaging materials. Since starch and organic acids are easily available and are low-cost materials, the authors do not doubt that starch modification with organic acids and their derivatives will attract even greater attention in future studies.
